# Hair Thread Tourniquet Syndrome in an Infant: Emergency Exploration Saves Limbs

**DOI:** 10.7759/cureus.6377

**Published:** 2019-12-13

**Authors:** Levin Kesu Belani, Juzaily F Leong, Parminder Singh Gill Narin Singh, Shalimar Abdullah

**Affiliations:** 1 Orthopaedic and Traumatology, Fakulti Perubatan, Universiti Kebangsaan Malaysia, Kuala Lumpur, MYS; 2 Hand and Microsurgery, Fakulti Perubatan, Universiti Kebangsaan Malaysia, Kuala Lumpur, MYS

**Keywords:** ischaemia, fingers, necrosis, hair, emergency, postpartum period

## Abstract

Hair thread tourniquet syndrome (HTTS) is a rare condition where fibres constrict around appendages causing ischaemia and necrosis. It is a sporadically reported condition, where almost all reported cases showed involvement of fingers, toes or genitalia. A significant number of the cases are infants aged two weeks to six months where it is attributed to the mother’s excessive hair fall due to hormonal changes after delivery. We present a two-month-old infant who was irritable for the past two days with her left ring finger exhibiting an ischaemic constriction with no apparent insulting agent. She successfully treated surgically after we suspected an incomplete removal of hair thread in the emergency department. We would like to highlight the importance of a high index of suspicion in cases as such as early intervention saves the appendage.

## Introduction

Hair thread tourniquet syndrome (HTTS) is an uncommon condition in which an appendage is constricted by a fibre leading to ischaemia and subsequently necrosis. The annual incidence of HTTS is 0.02%, and it presents as an emergency [1]. Attending doctors must have a high index of suspicion especially in children who are crying with no apparent reason as this diagnosis is poorly recognised. Delay in diagnosis and treatment can lead to amputation of the affected appendages. Mat Saad et al. reported that 44.2% of HTTS involved the penis, 40.4% involved the toes and 8.6% involved the fingers [2]. 

One of the possible insulting agents to cause HTTS is human hair, which is known to have a tensile strength greater than 29,000 pounds per square inch. Given its nature that expands when wet and tightens when dry, the human hair is capable of constricting any appendage of the body constricting blood vessels that may lead to a deadly appendage [3]. 

## Case presentation

**Figure 1:**
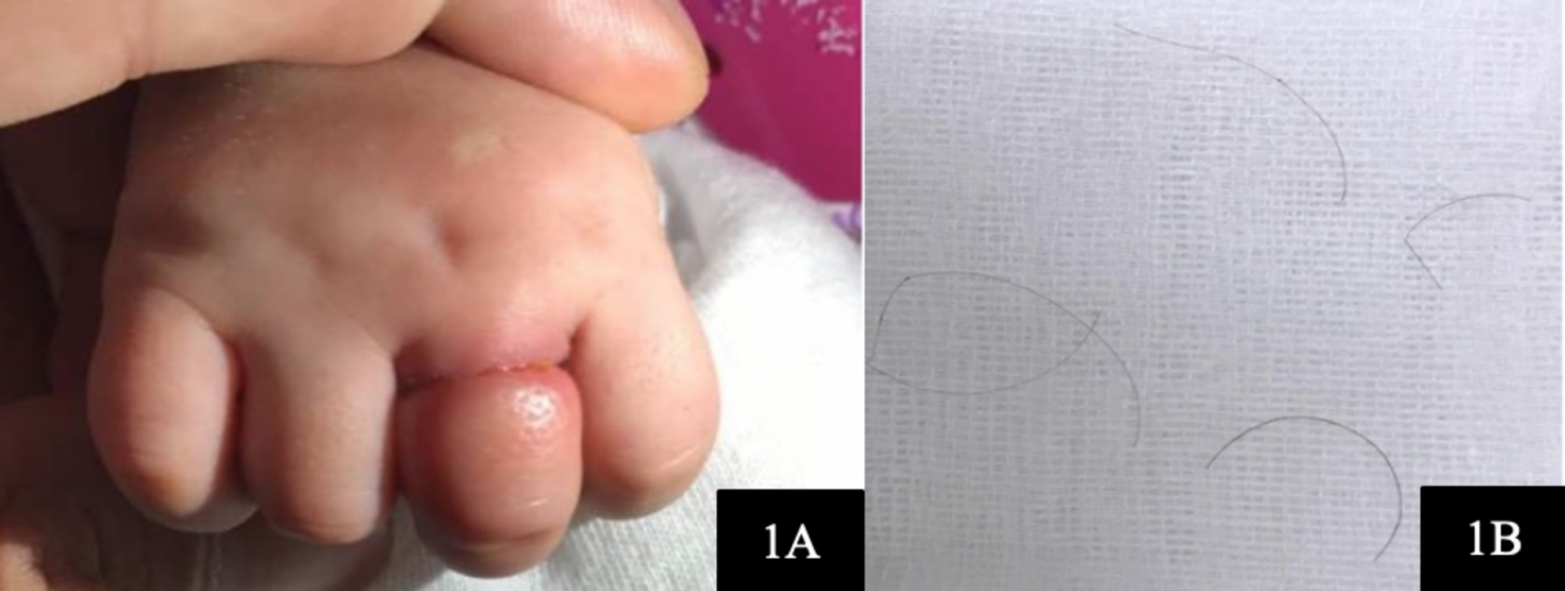
Strangulation over the proximal phalanx of the left ring finger causing oedema and congestion (1A). Fine hair strands removed using loupes in the emergency department (1B).

A two-month-old infant presented with persistent irritability despite all efforts to comfort her after one day, when the mother noted there was a swelling and constriction of her left ring finger upon removing her mitten. Physical examination revealed marked oedema, redness and congestion distal to the metacarpophalangeal joint of the ring finger (Figure 1A). There were no signs of tissue necrosis, no recent infections, no past medical history, and no known congenital issues such as constriction band syndrome and trauma. HTTS was considered, but no obvious constricting thread could be visualised.

We explored the finger in the emergency department using loupes (3x magnification) and a pen torch. A few fine hair strands were seen strangulating just distal to the metacarpophalangeal joint and cut free (Figure 1B). Although the perfusion of the finger was good, the congestion of the finger was only slightly reduced. The constricting mark was still very obvious, and we were not convinced that all the hair threads had been successfully removed. Subsequently, we explored the finger in the operating theatre under general anaesthesia as the child was fretful and moving.

**Figure 2:**
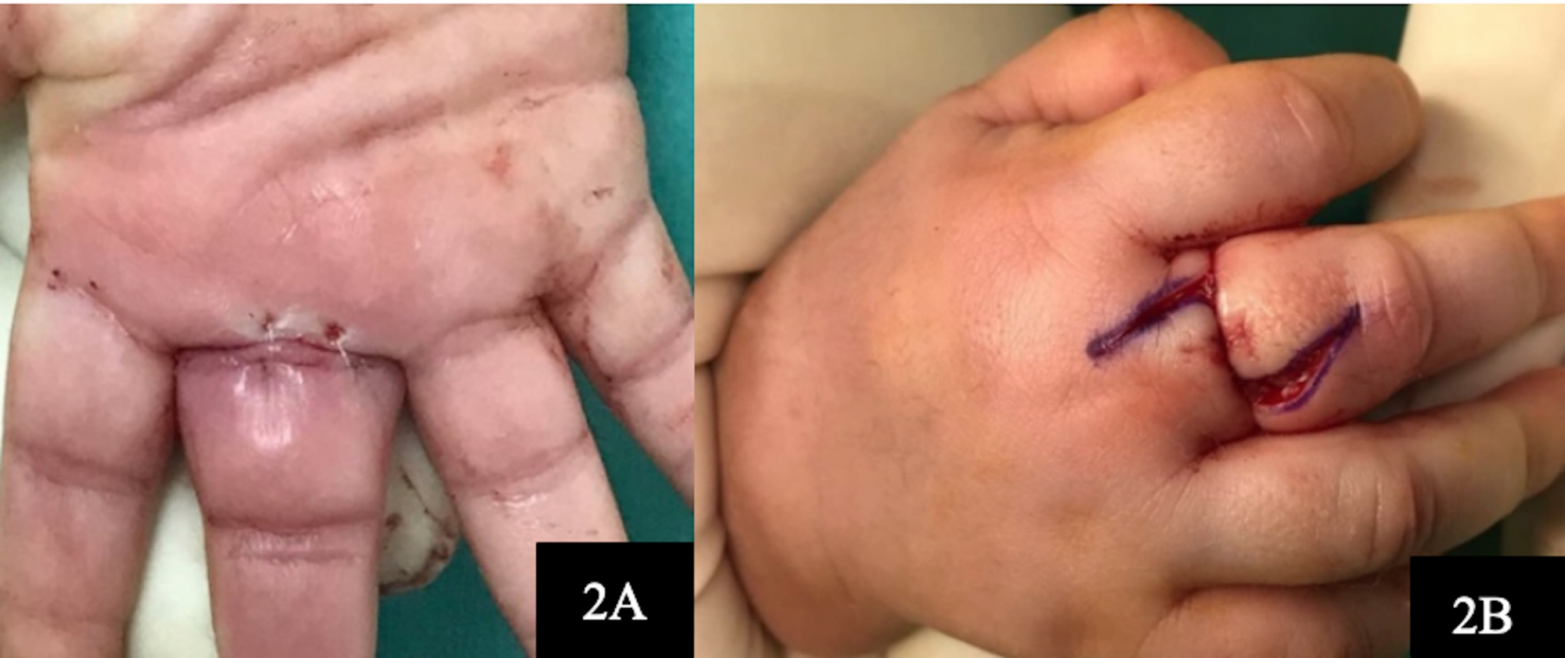
Undermining of the constricted soft tissues volarly with closure utilizing vertical interrupted sutures (2A). Exploration of the dorsal side of the middle finger with Z-plasty technique (2B).

Using the microscope in the operating theatre, we confirmed that there were no more hair threads detected. As the hair strand had strangulated the finger, and since we wanted to reduce the oedema, we decided to undermine the constricting mark and close it with a vertical mattress suture (Figure 2A, 2B). The oedema subsequently reduced and finger circulation was monitored for one day before the infant was allowed discharge. At three weeks, the wound over the finger had healed and the infant was actively moving the affected finger.

## Discussion

The aetiology of HTTS is currently not well known. However, the risk of getting HTTS is increased in an infant aged two weeks to six months due to telogen effluvium. Telogen effluvium is pronounced following pregnancy due to the endocrine flux, which leads to an excessive hair loss experienced by mothers in the post-partum period. This condition poses a higher chance of the hair strangulating the appendage as in the case above [4]. Another hypothesis of HTTS is that most appendages including the finger and toes are mostly covered in mittens, which are frequently washed without turning inside out. The accumulation of hair strands in these mittens could lead the infant winding these fibres around their appendages when they move their hands or legs freely in the mittens increasing the risk of HTTS [4]. As the hair may be deeply embedded, most patients with HTTS usually present three to four days later [5].

Although most cases are accidental, child abuse cannot be excluded in cases that involved multiple appendages and ecchymoses noted on the infant's body [4]. Other differential diagnosis that should be considered includes ainhum, pseudoainhum, infection, foreign body, insect bites and congenital constriction band [5].

The outcome of the affected appendage largely depends on the prompt detection, correct diagnosis and early intervention to completely remove any encircling fibres to restore the circulation. This can be done with the aid of a magnifying glass in the emergency setting or in the operating theatre. When the appendages are severely edematous, it is difficult to determine whether the constriction has been removed completely. This is exemplified in our case, where we had difficulty confirming complete removal of all hair strands due to the oedema. Surgical exploration is mandatory if a complete removal cannot be achieved or satisfactorily confirmed and should be done under general anaesthesia to allow optimal exploration. In this case, the Z-plasty approach was used in the exploration to prevent potential risk of scar contracture which may occur.

If the constricting agent is hair, the use of a depilatory agent is possible with minimal discomfort to the child [6]. However, it may not be suitable in cases where the hair is deeply embedded and unable to be visualised [7].

Education is to be targeted to both parents and attending doctors to raise awareness of this uncommon diagnosis. Parents should be advised to launder their children clothes inside and to avoid using coverings over their children extremities for a long period of time without supervision [8].

## Conclusions

HTTS is an uncommon occurrence. We should have a high index of suspicion as early diagnosis is vital in determining the outcome of the appendage. Early intervention either by non-surgical or surgical method to release the strangulation of the affected appendage is important to prevent ischaemic necrosis.
